# Identification of thiostrepton as a novel therapeutic agent that targets human colon cancer stem cells

**DOI:** 10.1038/cddis.2015.155

**Published:** 2015-07-02

**Authors:** S-Y Ju, C-YF Huang, W-C Huang, Y Su

**Affiliations:** 1 Institute of Biopharmaceutical Sciences, National Yang-Ming University, Taipei, Taiwan

## Abstract

Accumulating evidence shows that colorectal cancer stem cells (CRSCs) are largely responsible for the metastasis and relapse of colorectal cancer (CRC) after therapy. Hence, identifying new agents that specifically target CRSCs would help improve the effectiveness of current CRC therapies. To accelerate identification of agents targeting CRSCs, the Connectivity Map (CMap) approach was used. Among the top-ranked candidates, thiostrepton, a thiazole antibiotic, was selected for further investigation because of its known tumoricidal activity. Thiostrepton could selectively induce apoptosis in CRSC subpopulations in both parental HCT-15 and HT-29 human CRC lines as well as in EMT and chemoresistant clones derived from them. Further, we investigated its inhibitory effects on the sphere- and colony-forming capabilities of the aforementioned CRC lines. The *in vitro* inhibition of sphere and colony formation was associated with downregulation of various modulators of the stem cell phenotype. The combination of thiostrepton and oxaliplatin eradicated both CD44^+^ HCT-15 and HT-29 cells more efficiently than either drug alone. FoxM1, an oncogenic transcription factor, was identified as a critical positive modulator of stemness and as the main target of thiostrepton in the CRC lines. This is the first report showing the selective killing of CRSCs by thiostrepton, which has been proposed to be a promising anti-neoplastic agent. On the basis of its synergism with oxaliplatin in killing CRSCs *in vitro*, if this activity is confirmed *in vivo*, thiostrepton may be a promising agent to be used clinically in combination with current chemotherapies to improve the efficacy of these regimens.

Colorectal cancer (CRC) is the third most common cancer and the fourth leading cause of cancer-associated deaths globally,^1^ and this mortality is primarily attributed to its high incidence of metastasis.^2^ CRC has a stem cell (SC) subpopulation, which is generally considered to be the source of CRC tumor metastasis.^3, 4^ Colorectal cancer SCs (CRSCs) were identified as a rare group of CD133^+^ cells present in tumors resected from patients that had high *in vivo* tumorigenicity in immunodeficient animals.^5, 6^ Other markers such as CD44, CD166, CD26, EpCAM and aldehyde dehydrogenase (ALDH) have also been found to be useful in enriching CRSCs from tumor tissues as well as established CRC cell lines.^7, 8, 9, 10^ CRSCs have also been isolated as a ‘side population' by fluorescence-activated cell sorting owing to their expression of high levels of ABC-transporters which increases their ability to exclude hydrophobic dyes.^11^ Additionally, CRSCs have been identified in tumor spheres formed under defined culture conditions *in vitro*.^12^ Finally, recent studies have demonstrated a convincing link between epithelial-to-mesenchymal transition (EMT) and CSCs as well as the association of these processes with CRC progression and therapeutic resistance.^13, 14^

As the essential roles of CRSCs in therapeutic resistance of CRC have been characterized, strategies targeting this subpopulation have been developed to improve therapeutic outcomes for CRC patients. One of the methods that can be used to identify CRSC targets is by evaluating signaling pathways specific to CSC self-renewal and/or maintenance of stemness and conduct studies to analyze the potential of diminishing these new targets for effective eradication of CRSCs. On the basis of this strategy, novel leads worthy of further investigation as potential CRC drugs have been found by targeting Wnt, Hedgehog, and Notch pathways.^15, 16^ For example, LGK974, a specific inhibitor for Porcupine, a membrane-bound O-acyl- transferase required for palmitoylation and secretion of Wnt ligands has been shown to suppress *in vitro* and *in vivo* growth of Wnt-dependent tumor cells.^17^ Vismodegib, a Hedgehog pathway inhibitor, has been approved by the US FDA for treating certain forms of basal cell carcinoma (for a review, see Sandhiya *et al.*^18^). Moreover, antitumor efficacy and prolonged disease stabilization has been observed in patients with advanced solid tumors co-treated with RO4929097, a *γ*-secretase/Notch signaling pathway inhibitor, and cediranib, a VEGF receptor tyrosine kinase inhibitor.^19^ Strategies specifically targeting CSC surface markers such as CD44 have been explored successfully in leukemia and breast cancer in pre-clinical models.^20, 21^ Additionally, salinomycin, identified originally as a drug with selective toxicity against breast CSCs,^22^ was shown to be effective against a variety of other CSCs.^22^ Other studies have demonstrated that IGF-1 inhibition,^23^ HER-2 signaling blocking,^24^ IL-4 neutralization,^25^ or BMP4 addition^26^ inhibit *in vivo* tumorigenicity of CRSCs and the latter two treatments can further sensitize CRSC-derived tumors to conventional chemotherapeutic agents. Recently, mTOR suppression by rapamycin or PP242 has been shown to decrease stemness properties as well as suppress the increase in CSC populations that can be induced by 5-FU and oxaliplatin treatment of CRC cells.^27^ Despite the discovery of these promising agents, more drugs (especially the ones already approved for clinical use) are still needed to expand the arsenal of agents that can combat this rare but deadly subpopulation.

To identify new compounds, we used the Connectivity Map (CMap) approach, which has been shown to be effective for drug discovery and development.^28^ Among several top-ranked candidates identified, thiostrepton, a thiazole antibiotic, was selected for further investigation because of its well-documented tumoricidal activity.^29, 30^ In the present study, we demonstrated that thiostrepton selectively kills the SC subpopulations in HCT-15 and HT-29 human CRC lines as well as EMT and chemoresistant clones derived from them. Current work also showed that thiostrepton triggered apoptosis in HCT-15 and HT-29 CRSCs and inhibited colony and sphere formation, with downregulation of various stemness regulators. Interestingly, synergistic killing effects with thiostrepton and oxaliplatin were noted against HCT-15 and HT-29 cells as well as their SC subpopulations. Finally, we identified FoxM1 as a critical positive modulator of stemness in the aforementioned CRC lines and the main target of thiostrepton. Together, these findings warrant further investigation of the therapeutic potential of thiostrepton as an adjuvant treatment with CRC chemotherapy once its *in vivo* antitumor efficacy is confirmed.

## Results

### Thiostrepton reduces the proportion of CRSCs

**Figure 1:**
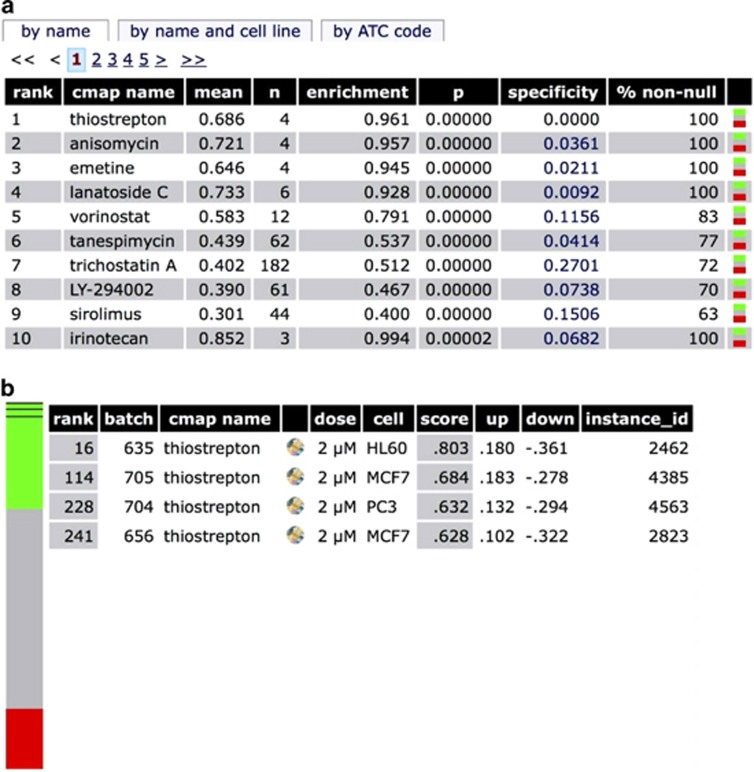
Identification of thiostrepton as a potential anti-cancer SC agent. (**a**) The top 10 candidates with a positive enrichment score were shown here. The ‘mean' stands for the average connectivity scores of microarray profiles of various instances (e.g. HL60 cell line treated with thiostrepton) and the ‘*n*' stands for the number of microarray profiles of a drug (e.g. thiostrepton). The ‘enrichment' score was ranged from 1 to −1 and delivers the enrichment of the instances of a drug among the 6100 instances of all drugs. (**b**) The colored bar with three colors represents 6100 instances in the CMap 2.0 database. Green means positive connectivity, red means negative connectivity, and gray means no connectivity. The dark lines in the green zone of the bar represent the instances of thiostrepton. It shows that thiostrepton is ranked as no.16, 114, 228, and 241 compared with the other instances in the CMap 2.0 database

**Figure 2:**
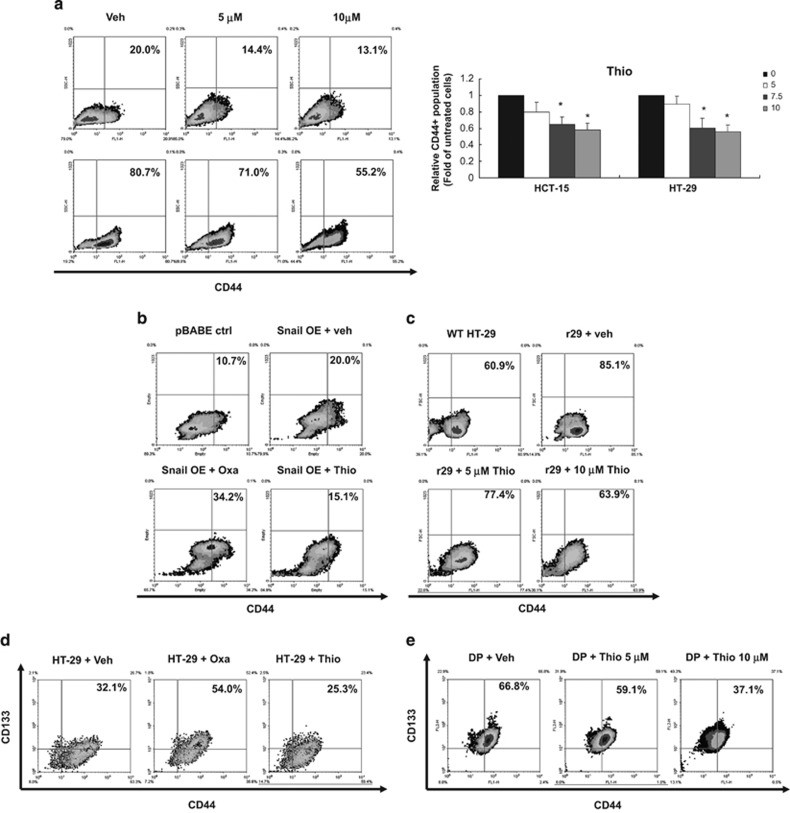
Thiostrepton reduces SC subpopulations in HCT-15 and HT-29 human colon cancer lines. (**a**) HCT-15 and HT-29 cells were treated with dimethyl sulfoxide (DMSO as a vehicle control, Veh) or the indicated concentrations of thiostrepton for 48 h before flow cytometry was used to measure the percentage of the CD44^+^ subpopulation, which is indicated by the number shown in the upper-right of each quadrant. Data in the right bar graph are mean±S.D. from three independent determinations. *P<0.05 when compared with vehicle control by Student's *t*-test. (**b**) Vector control (pBABE ctrl) and Snail-overexpressing (Snail OE) clones established from HCT-15 cells were treated with DMSO (veh), oxaliplatin (Oxa, 25 *μ*M), and thiostrepton (Thio, 5 *μ*M) for 48 h before their CD44^+^ subpopulations were measured. (**c**) Wild-type (WT) and oxaliplatin-resistant (r29) HT-29 cells were treated with DMSO and the latter were incubated with 5 and 10 *μ*M thiostrepton for 48 h before their CD44^+^ subpopulations were analyzed. (**d**) Wild-type HT-29 cells were treated with DMSO, oxaliplatin, and thiostrepton as above described except their CD133^+^CD44^+^ (double positive) subpopulations were measured. (**e**) The remaining portions of the double-positive subpopulations in the freshly isolated CD133^+^CD44^+^ HT-29 cells were examined after they were incubated with DMSO, 5 and 10 *μ*M thiostrepton for 48 h

To identify drug candidates with selective toxicity against SC populations present in human CRC lines, we extracted an anti-CSC gene signature from the study of *Suvà et al.*^31, 32^ and used it as the input signature to query CMap 2.0. Among the top 10 drugs that shared positive connectivity with the anti-CSC signature, thiostrepton was ranked as the top one (Figure 1). MTT assays were then carried out to evaluate the cytotoxicity of thiostrepton on HCT-15, HT-29 and HCT-116 CRC lines. As shown in Table 1, thiostrepton killed CRC cells more efficiently than oxaliplatin, a first-line treatment for CRC. To investigate the effect of thiostrepton on human CRSCs, flow cytometry was used to assess its influence on cell surface expression of the CRSC marker CD44 in HCT-15 and HT-29 cells. As shown in Figure 2a, thiostrepton treatment reduced CD44^+^ subpopulations in both CRC lines. In contrast, a significant increase in these cells was observed after treatment with oxaliplatin (Supplementary Figure S1). Because earlier studies have shown that CSC subpopulations could be enriched by EMT induction^14, 33^ or incubation in suboptimal concentrations of cytotoxic drugs,^23, 34^ a Snail-overexpressing HCT-15 clone (Snail OE) and an oxaliplatin-resistant HT-29 subline (r29, Supplementary Figure S2) were established. As expected, 10 and 25% increases in CD44^+^ subpopulations were observed, respectively, for the cell lines (Figures 2b and c). These clones were then used to evaluate the efficacy of thiostrepton against CRSCs. In agreement with the above findings, thiostrepton reduced the CD44^+^ subpopulation in the Snail OE clone, while oxaliplatin treatment caused an increase in CD44^+^ cells in similar clone (Figure 2b). A dose-dependent decrease in the CD44^+^ subpopulation in r29 cells was also noted after thiostrepton treatment (Figure 2c). To confirm the toxicity of thiostrepton against CRSCs, flow cytometry was used to measure the proportion of the CD44 and CD133 double positive (CD44^+^/CD133^+^) HT-29 cells that remained after the parental cells were treated with oxaliplatin or thiostrepton, because these cells are considered CRSCs.^35^ The CD44^+^/CD133^+^ subpopulation in HT-29 cells was increased after oxaliplatin treatment and was decreased after thiostrepton incubation (Figure 2d). Additionally, the freshly isolated CD44^+^/CD133^+^ HT-29 cells were also effectively killed by thiostrepton (Figure 2e).

### Thiostrepton induces apoptosis of CRSCs

**Figure 3:**
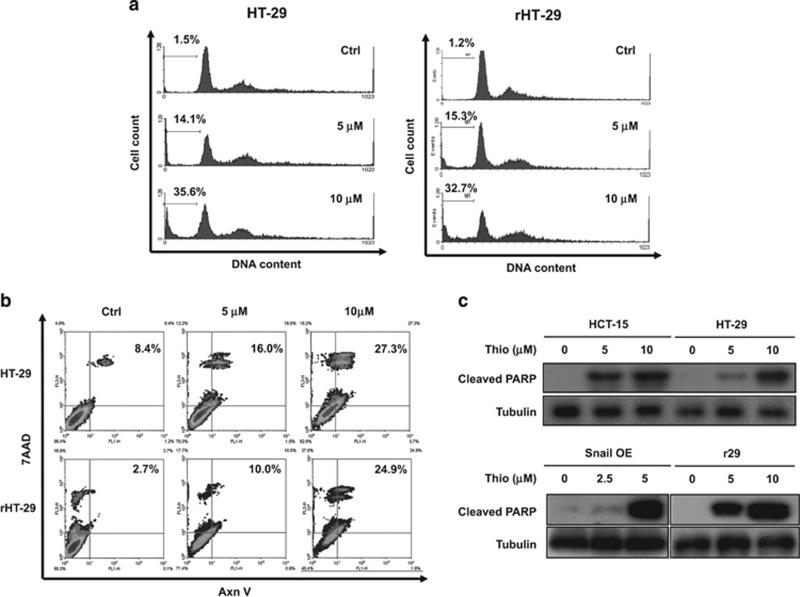
Thiostrepton induces apoptosis of SCs present in HT-29 and HCT-15 lines. (**a**) HT-29 and r29 cells were treated without (Ctrl) or with 5 and 10 *μ*M thiostrepton for 48 h before their DNA contents were analyzed by flow cytometry. The percentage of the sub-G1 populations were indicated by the numbers shown on the left of each panel. (**b**) Both HT-29 and r29 cells after being treated with vehicle (Ctrl) and 5 or 10 *μ*M thiostrepton for 48 h were collected for annexin V and 7-AAD staining. The apoptotic populations (numbers in the top right quadrant) were analyzed by flow cytometry. (**c**) Total lysates prepared from parental and Snail-overexpressing (Snail OE) HCT-15 cells as well as wild-type HT-29 and r29 cells after they were treated with or without the indicated concentrations of thiostrepton for 48 h were subjected to immunoblot analysis using an antibody against cleaved PARP as a probe. Tubulin signal served as a loading control

Because thiostrepton has been reported to inhibit growth and induce apoptosis in various human cancer cells,^30, 36, 37^ the effect of this drug on regular as well as SC-enriched CRC cells was next investigated. As can be seen, a dose-dependent increase in the sub-G1 fraction in HT-29 and r29 cells was observed after thiostrepton treatment (Figure 3a). Annexin V and 7-AAD double-positive apoptotic populations in the two CRC lines were also increased in a dose-dependent manner by this drug (Figure 3b). Furthermore, apparent cleavage of PARP in HCT-15 and its Snail-overexpressing clone as well as in HT-29 and r29 cells was detected after thiostrepton treatment (Figure 3c). These results clearly demonstrated that thiostrepton induces apoptosis of CRSC-enriched clones derived from both HCT-15 and HT-29 cells.

### Thiostrepton suppresses sphere and colony formation, as well as the expression of some stemness-related proteins in human CRSCs

**Figure 4:**
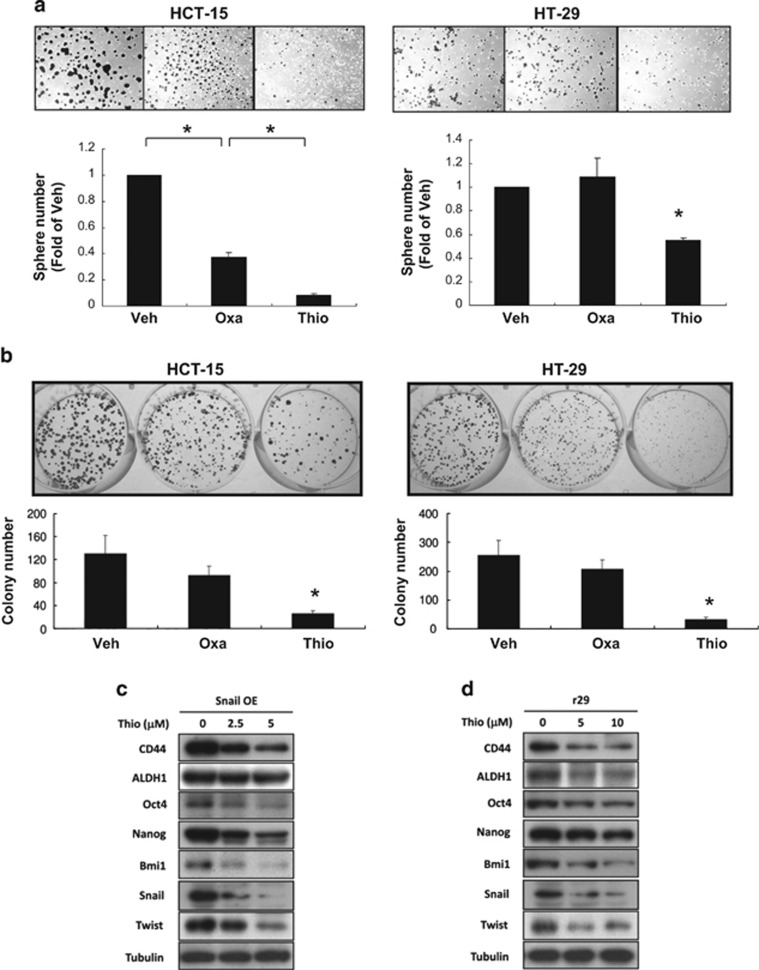
Thiostrepton suppresses tumor sphere- and colony-formation potentials of HCT-15 and HT-29 cells as well as the expression of factors involved in stemness maintenance. (**a**) HCT-15 and HT-29 cells were cultured in defined media supplemented continuously with DMSO (Veh), oxaliplatin (Oxa, 25 *μ*M), or thiostrepton (Thio, 5 *μ*M). Seven days later, spheres stained by MTT were photographed ( × 40) and counted using software. *P<0.05 when compared with the DMSO-treated cells by Student's *t*-test. (**b**) Clonogenic assays were performed using cells exposed previously to DMSO (Ctrl), oxaliplatin, or thiostrepton for 6 h as described in ‘Materials and Methods'. Ten days after seeding, colonies were stained by crystal violet and their numbers were counted using Colony 1.1 software. *P<0.05 when compared with the DMSO-treated cells by Student's *t*-test. Total lysates prepared from (**c**) Snail OE and (**d**) r29 cells after being treated with the indicated doses of thiostrepton for 48 h were subjected to western blot analyses using antibodies against CD44, ALDH1, Oct4, Nanog, Bmi1, and Twist as probes. Tubulin signal served as a loading control

Because thiostrepton reduced CD44^+^ subpopulations present in human CRC lines, we assessed whether other relevant phenotypes for CRSCs were also suppressed by this antibiotic. Sphere formation of HCT-15 and HT-29 cells in the continuous presence of thiostrepton or oxaliplatin was examined. The degree of tumor spheres formed by both CRC lines was decreased by thiostrepton treatment. A marked reduction in HCT-15 tumor sphere formation after oxaliplatin treatment was also noted (Figure 4a). The influence of short exposure of thiostrepton on CRSCs was evaluated by colony formation of HCT-15 and HT-29 cells after they were treated with oxaliplatin and/or thiostrepton for 6 h prior to seeding. As shown in Figure 4b, while oxaliplatin treatment of both CRC lines only reduced the size of colonies, short exposure to thiostrepton drastically reduced their colony numbers. These results suggested that thiostrepton, unlike oxaliplatin, was able to eradicate clonogenic subpopulations present in these human CRC cell lines. To elucidate the molecular mechanisms underlying the suppressive effects of thiostrepton on CRSC self-renewal, immunoblotting was used to analyze the protein levels of various markers of stemness, including CD44, ALDH1, Oct4, Nanog, Bmi1, Snail, and Twist in Snail OE and r29 clones after thiostrepton treatment. To no surprise, protein levels of all the aforementioned factors were decreased by thiostrepton (Figures 4c and d). Because earlier studies have reported that some of the anticancer effects of thiostrepton could be attributed to its proteasome inhibitory activity,^38^ we assessed the contribution of this activity to the stemness-suppressing effects of thiostrepton by evaluating the stemness properties of HCT-15 and HT-29 cells after treatment with the proteasome inhibitors MG132 and Carfilzomib (Kyprolis). Strikingly, neither inhibitor decreased the CD133^+^CD44^+^ subpopulations in HCT-15 and HT-29 cells (Supplementary Figure S3). Moreover, despite the reduction in protein levels for stemness markers such as Oct4, Nanog, and Bmi1 by both inhibitors, neither agent decreased SC marker protein levels in the CRC lines as effectively as thiostrepton (Supplementary Figure S4). Collectively, the above findings suggested that the proteasome inhibitory activity of thiostrepton is not a key contributor to its stemness-reducing effects on CRSCs.

### Thiostrepton synergizes with oxaliplatin in killing CRSCs

**Figure 5:**
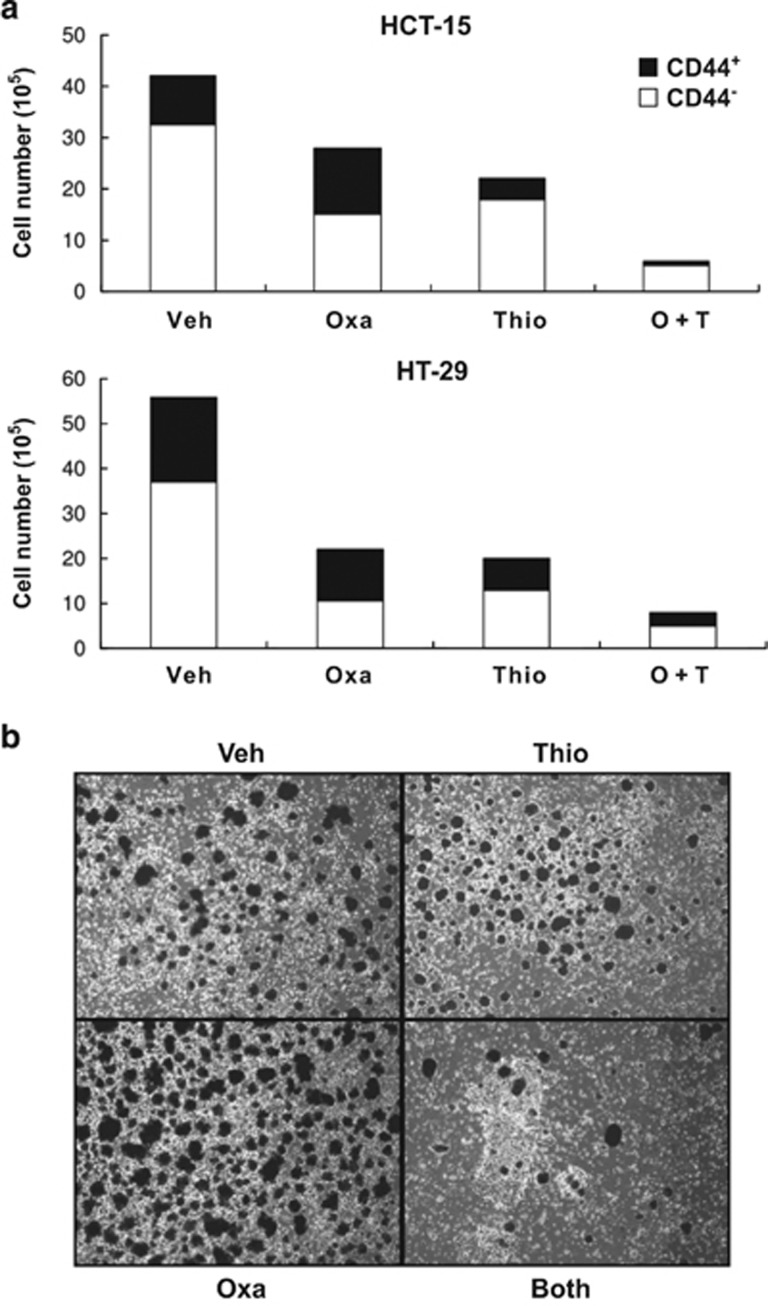
Thiostrepton acts synergistically with oxaliplatin to kill CD44^+^ subpopulations in HCT-15 and HT-29 cells as well as suppress sphere formation of HCT-15 cells. (**a**) HCT-15 and HT-29 cells were treated with DMSO (Veh), oxaliplatin (Oxa, 25 *μ*M), or thiostrepton (Thio, 5 *μ*M), or both drugs together for 48 h. The number of viable CD44^+^ (black) and CD44^−^ (white) cells separated by flow cytometry were counted by dye exclusion assays. (**b**) Spheres formed from HCT-15 cells cultured in defined media containing DMSO (Veh), 15 *μ*M oxaliplatin (Oxa), 5 *μ*M thiostrepton (Thio), or both drugs (Both) for 5 days were stained with MTT and then photographed under a microscope (X40)

Having demonstrated that thiostrepton alone could reduce the SC subpopulations in HCT-15 and HT-29 cells, we asked what effect the combination of thiostrepton with oxaliplatin would have on CRC cell killing, especially the CD44^+^ subgroups. As can be seen, while at least 45% of surviving cells were CD44^+^ after oxaliplatin treatment, CD44^+^ ones accounted for less than 15% and 30% of the viable HCT-15 and HT-29 cells, respectively, after being incubated with thiostrepton. Even greater reductions in CD44^+^ cells in HCT-15 and HT-29 lines were detected when the two drugs were combined (Figure 5a). Also, established tumor cell spheres generated from HCT-15 cells were reduced by treatment with this drug combination even though sphere growth was enhanced by oxaliplatin alone (Figure 5b). These results demonstrated synergism between thiostrepton and oxaliplatin for the eradication of CRSCs.

### FoxM1 expression in HCT-15 and HT-29 cells is suppressed by thiostrepton and this antibiotic inhibits CD44^+^ HCT-15 cells

**Figure 6:**
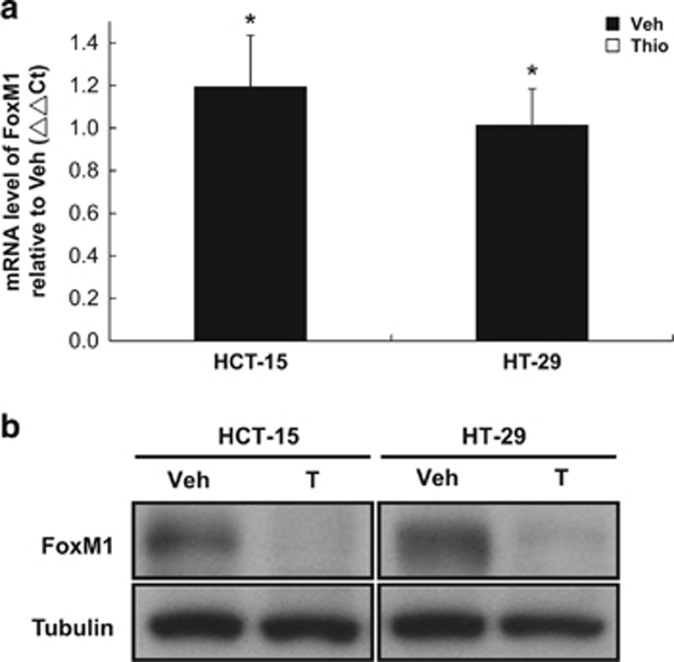
Thiostrepton suppresses mRNA and protein levels of FoxM1 in HCT-15 and HT-29 cells. (**a**) FoxM1 mRNA levels were determined by real-time RT-PCR using total RNA prepared from HCT-15 and HT-29 cells after being treated with 5 *μ*M thiostrepton for 6 h. (**b**) FoxM1 protein levels were evaluated in the same cells after they were incubated with 5 *μ*M thiostrepton for 48 h by western blotting. Tubulin signal was used as a loading control. Data representing the mean±S.D. of three independent experiments were analyzed by Student's *t*-test (*P<0.05)

**Figure 7:**
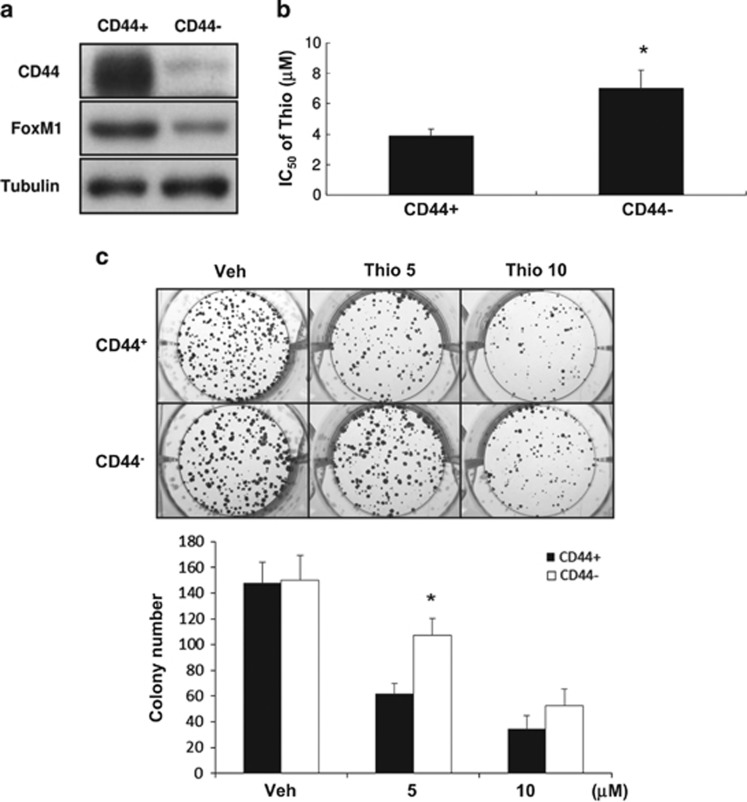
Higher expression of FoxM1 is found in CD44^+^ HCT-15 cells, which are more sensitive to thiostrepton. (**a**) Protein levels of CD44 and FoxM1 in CD44^+^ and CD44^−^ HCT-15 cells were analyzed by western blotting. Tubulin signal was used as a loading control. (**b**) The IC_50_ values of thiostrepton on CD44^+^ and CD44^−^ HCT-15 cells were determined as described. *P<0.05 by Student's *t*-test. (**c**) A colony-formation assay was carried out by treating cells with DMSO (Veh) or 5 or 10 *μ*M thiostrepton for 6 h before their washout of drug or control. Cells were cultured in regular media for 10 more days and colonies were stained with crystal violet. Data representing the mean±S.D. of three independent experiments were analyzed by Student's *t*-test (*P<0.05)

FoxM1 is a transcription factor key to malignant progression of various tumors^39^ with the demonstrated roles of FoxM1 in stemness, chemoresistance, invasivness, EMT,^40, 41^ and the maintenance of self-renewal capacity of certain types of SCs.^42, 43^ In previous studies, FoxM1 was shown to be targeted by thiostrepton.^44, 45^ We thereby assessed whether FoxM1 was affected by thiostrepton in HCT-15 and HT-29 cell lines. In agreement with the earlier observations made by others, mRNA levels of FoxM1 in these cells drastically decreased after a short exposure (6 h) to thiostrepton (Figure 6a), subsequently, reduced protein level could be observed after 48 h treatment (Figure 6b). These results support FoxM1 as the main target of this antibiotic in human CRC cells because direct transcription suppression on *FoxM1* is the most likely explanation for the effects observed after short-term treatment with this thiazole drug. We examined whether HCT-15 SC subpopulations (CD44^**+**^) expressed elevated levels of FoxM1, possibly affecting their response to thiostrepton. Higher FoxM1 protein levels were found in CD44^+^ HCT-15 cells (Figure 7a), and this subpopulation was more susceptible to thiostrepton (Figure 7b). After short-term (6 h) treatment with this drug, CD44^+^ HCT-15 cells formed colonies at a slower rate and they were smaller (Figure 7c), demonstrating the selective cytotoxicity of thiostrepton against CRSCs. These data indicate that elevated expression of FoxM1 in HCT-15 CRSCs may account for their increased sensitivity to thiostrepton.

### FoxM1 is required for maintenance of the CS subpopulation and the susceptibility of CRSCs to thiostrepton in HT-29 and HCT-116 cells

**Figure 8:**
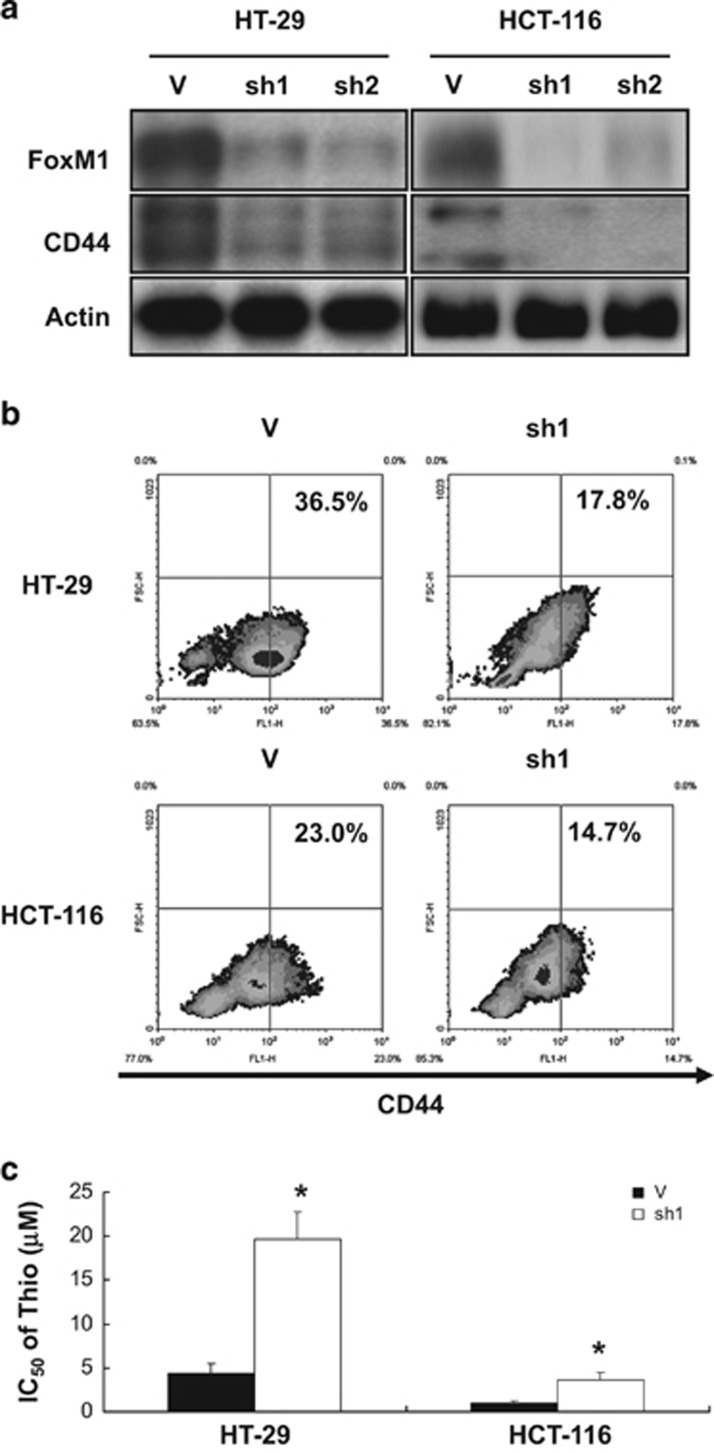
Knockdown of FoxM1 expression in HT-29 and HCT-116 cells reduces their CD44^+^ subpopulations and their sensitivity to thiostrepton. Vector-control and FoxM1-knockdown clones were established from HT-29 (29 V, 29sh1 and 29sh2) and HCT-116 (116 V, 116sh1, and 116sh2) cells by retroviral infection. (**a**) The protein levels of FoxM1 and CD44 in the aforementioned clones were analyzed by western blotting. Actin signal served as a loading control. (**b**) The percentage of CD44^+^ subpopulations (numbers in the top right quadrant) in 29 V and 29sh1 clones and 116 V and 116sh1 cells were measured by flow cytometry. (**c**) The IC_50_ values of thiostrepton on the aforementioned four clones were determined as described in the ‘Materials and Methods'. *P<0.05 when compared with their respective vector control clones by Student's *t*-test

Having found that FoxM1 may positively modulate the stemness in HCT-15 cells and be the primary target mediating their sensitivity to thiostrepton, we dissected the role of this transcription factor in the maintenance of CD44^+^ subpopulations in two other CRC lines as well as evaluating their response to thiostrepton. Significant decreases in CD44 expression levels were found in the FoxM1-knockdown clones derived from HT-29 and HCT-116 cells (Figure 8a). Additionally, the CD44^+^ subpopulation in the above-mentioned FoxM1-knockdown clones was also reduced (Figure 8b). Consistent with these results, the sensitivity of these knockdown clones to thiostrepton was reduced in comparison with vector control cells (Figure 8c). These results suggested that FoxM1 is not only responsible for maintaining key stemness phenotypes of the CRC lines evaluated but also is the primary target for thiostrepton.

## Discussion

To improve the efficacy of current treatments for CRC, novel agents that can eradicate the tenacious CRSCs present in patients need to be identified and developed. In this study, our clear demonstration that thiostrepton could synergize with oxaliplatin, a widely used CRC chemotherapeutic agent, in killing CRSCs, strengthens the rationale for its clinical usage in CRC treatment.

To characterize the potential target of thiostrepton in CRSCs, we focused on Forkhead box M1 (FoxM1), an oncogenic transcription factor critical for various cellular functions including self-renewal of normal SCs,^39, 46, 47, 48^ because the inhibition of this factor was responsible for the apoptotic effects of thiostrepton and another thiazole antibiotic, siomycin A.^30, 49^ In earlier studies, FoxM1 was proposed to be a promising therapeutic target for cancers because upregulated expression of this protein was found in most human solid tumors including CRC.^50^ Subsequently, crucial roles played by FoxM1 in promoting tumor initiation,^51^ invasion,^52^ and metastasis^53, 54^ have also been reported. More interestingly, the involvement of this factor in modulating signaling pathways crucial for SC development has recently been reported by others. For example, FoxM1 is not only required for pluripotency maintenance of P19 embryonal carcinoma cells by regulating Oct4,^43^ but also enhances tumorigenesis of glioblastoma cells by promoting the nuclear translocation of β-catenin, a critical mediator of Wnt signaling, *via* a direct protein-protein interaction.^55^ Taken together, these findings support our data that the cytotoxic effect of thiostrepton on CRSCs is due to higher expression of FoxM1 in these cells and explain the decreases in both SC subpopulations and the susceptibility to this antibiotic observed in FoxM1-knockdown clones. However, chemosensitization resulting from FoxM1 silencing in other tumor cell types has also been reported.^56, 57^ More work is needed to resolve these discrepancies.

Because enrichment of CSCs from various types of cancers after treatment with conventional therapeutic agents has been demonstrated,^33, 34, 58^ strategies that reduce the expression of stemness-related genes were proposed as a way to overcome drug resistance. In this regard, the nuclear factor-erythroid 2 p45-related factor 2 (Nrf2)/antioxidant response element (ARE) pathway has been shown to be the plausible cause for the resistance of HT-29 cells to 5-FU treatment. Accordingly, silencing of Nrf2, a newly identified pluripotency gene,^54^ not only inhibited the expression of its target genes involved in cytoprotection but also increased 5-FU cytotoxicity.^59^ Moreover, the chemosensitization of HCT-116 cells to 5-FU by curcumin, a plant polyphenol, has recently been attributed to its ability in suppressing CSC pools.^59^ Consistent with these findings, the CD44^+^ subpopulations in HCT-15 and HT-29 cells were dramatically increased after oxaliplatin treatment (Supplementary Figure S1), so was the sphere-forming ability of the former (Figure 5b). On the contrary, combined treatment with oxaliplatin and thiostrepton markedly diminished not only the CD44^+^ subpopulations in two CRC lines but also HCT-15 sphere formation (Figure 5). Collectively, our data suggest that thiostrepton, *via* uncharacterized mechanisms, reverses the SC-enriching effect of oxaliplatin and sensitizes CRSCs to this commonly used anti-CRC drug.

In conclusion, thiostrepton was identified as a novel agent with selective *in vitro* cytotoxicity against CRSCs present in HCT-15, HT-29, and HCT-116 cell lines. However, to enable the clinical use of this thiazole antibiotic as an adjunct in CRC treatment, animal studies are required to assess the *in vivo* efficacies of thiostrepton alone or in combination with oxaliplatin and/or other clinically available agents in suppressing tumor growth and tumor relapse after treatments are stopped as well as the safety of this drug. Continued research to dissect the cytotoxic mechanisms of thiostrepton on CRSCs may allow us not only to identify more novel target(s) in this rare subpopulation but also to design better treatments for CRC.

## Materials and Methods

### Anti-CSC signature

Differentially expressed probe sets whose adjusted *P*-value≤0.05 and abs (fold change)≥2 were selected as the anti-CSC signature by using GEO2R.^60^ The up and down probe sets were used as the input signature and were uploaded to CMap 2.0 website (https://www.broadinstitute.org/cmap/).^31^

### Cell culture

HCT-15, HCT-116, and HT-29 human colon carcinoma cell lines were purchased from the American Type Culture Collection (ATCC, Manassas, VA, USA). The parental lines were maintained in RPMI-1640 medium supplemented with 10% fetal calf serum (Biological Industries, Kibbutz Beit Haemek, Israel), 100 units/ml penicillin, 100 *μ*g/ml streptomycin, and 25 *μ*g/ml amphotericin B (Biological Industries, ATCC) at 37 °C in 5% CO_2_. Stable transfectants derived from them (described below) were maintained under similar conditions except that the appropriate antibiotics were added to the media. To generate an HT-29 line resistant to oxaliplatin, cells were maintained in regular medium containing 5 *μ*M oxaliplatin for 2 days followed by incubation in medium without drug for another 2 days. After three rounds of drug selection, an MTT assay was carried out to confirm that the IC_50_ of oxaliplatin for the surviving cells, designated as rHT-29 line, was much greater than that of the parental cells (see below).

### Chemical

Thiostrepton obtained from Sigma-Aldrich (Taufkirchen, Germany) was dissolved in 100% DMSO at a final concentration of 10 mM as a stock solution. The working concentration of thiostrepton was either 5 or 10 *μ*M with a less than 0.1% final concentration of DMSO throughout this study.

### Virus preparation and generation of stable clones

The Snail expression vector was a kind gift from Dr Chiou S.H. (Institute of Pharmacology, National Yang-Ming University, Taipei, Taiwan, R.O.C.). FoxM1-knockdown stable clones were generated according to a previously described protocol.^61^ Briefly, two pLKO.1 lentiviral vectors containing different shRNA sequences (5′-CGCTACTTGACATTGGACCAA-3′ designated as sh1; 5′-GCCAATCGTTCT CTGACAGAA-3′ designated as sh2) targeting FoxM1 mRNA were purchased from the National RNAi Core Facility Platform (Nankang, Taipei) and were used to generate recombinant virus for infection. A Snail-overexpressing HCT-15 clone (Snail OE) was established by selecting virus-infected cells in a medium containing 100 *μ*g/ml hygromycin. FoxM1-knockdown clones from HCT-116 (sh1 and sh2) and HT-29 (sh1 and sh2) cells were generated by a similar protocol except that infected cells were selected in 1.5 and 1 *μ*g/ml of puromycin, respectively.

### Isolation of CD44^+^ cells by fluorescence-activated cell sorting

After trypsinization, single cell suspension (1 × 10^7^) prepared in regular medium was incubated with an anti-CD44 antibody conjugated with FITC (Beckman Coulter Inc., Krefeld, Germany, IM1219U). CD44^+^ cells sorting was performed on a BD FACSAria (BD Biosciences Pharmingen, San Diego, CA, USA) using the index sorting function.

### Isolation of CD44/CD133 double positive cells and assay of apoptosis by fluorescence-activated cell sorting

Cells detached from dishes by TrypLE (Invitrogen, Carlsbad, CA, USA) treatment were resuspended at a density of 1 × 10^6^ cells/ml in complete media containing FITC-labeled anti-CD44 antibody alone or with PE-labeled anti-CD133 antibody (Miltenyi Biotec, Auburn, CA, USA, 130-080-801). After 1-h incubation on ice, flow cytometry was carried out to analyze and isolate the CD44^+^ and/or CD44^+^CD133^+^ (double positive, DP) subpopulations in HCT-15, HT-29, Snail OE, and r29 cells. Thiostrepton-triggered apoptosis of HT-29 and r29 cells was analyzed after drug (5 or 10 *μ*M) treatment for 48 h. Cells were harvested and incubated with 1 *μ*g/ml FITC-labeled Annexin V (Beckman Coulter Inc.) and 10 *μ*g/ml 7-amino-actinomycin D (7-AAD, AnaSpec, Fremont, CA, USA) in RPMI medium for 30 min on ice. Flow cytometry was performed to measure the percentage of apoptotic cells. To analyze the sub-G1 population induced by thiostrepton, drug-treated cells were fixed in cold 70% ethanol for 30 min on ice before being washed twice in PBS at room temperature. After RNase A (10 mg/ml, Sigma, St. Louis, MO, USA) treatment, cellular DNA was stained with 7-AAD and samples were then subjected to flow cytometry.

### MTT assays

Cells were seeded at a density of 1 × 10^4^ per well onto 96-well plates. After overnight culture, cells were treated with various drugs at the indicated concentrations for 48 h before 1 mg/ml MTT [3-(4,5-dimethylthiazol-2-yl)-2,5-diphenyltetrazolium bromide, Sigma] was added. Three hours later, medium was removed and formazan crystals were dissolved in isopropanol and the optical density of each sample was measured by an ELISA reader (Bio-Rad Laboratories, Hercules, CA, USA) at 570 nm. Cells cultured in the presence of DMSO (vehicle) alone were considered untreated control.

### Colony-formation and spheroid-formation assay

Colony-formation assays were conducted as described.^61^ Briefly, HCT-15 or HT-29 cells were seeded at a density of 1 × 10^3^ per well in six-well plates. Forty-eight hours later, cells were treated with oxaliplatin or thiostrepton for 6 h. Medium was replenished every 3 days. After 10 days, crystal violet staining was performed and the number of colonies was counted using Colony, version 1.1 software (Fujifilm, Tokyo, Japan). Tumor spheroids were generated as previously described.^62^ Briefly, single-cell suspensions of 1 × 10^4^ cells were seeded in P60 culture dishes precoated in a 50 *μ*g/ml poly-2-hydroxyethyl methacrylate (polyHEMA) solution (Sigma-Aldrich) overnight in 37 °C incubator. After 3 weeks of culture in defined media (RPMI 1640 (Gibco/Invitrogen, Karlsruhe, Germany) supplemented with 6 mg/ml glucose, 4 mg/ml BSA (Sigma-Aldrich), 10 ng/ml bFGF (Peprotech, London, UK), 20 ng/ml EGF (PeproTech) and 1 × N2 (Gibco/Invitrogen)), spheroids were stained and counted.

### Real-time RT-PCR

Real-time RT-PCR was performed primarily as described (Ju *et al.*, 2014).^61^ Briefly, total RNAs were extracted by Trizol (Invitogen) and reverse-transcribed using MMLV reverse transcriptase (Invitrogen). A MiniOpticon Real-Time PCR Detection System (Bio-Rad Laboratories) was used to carry out SYBR Green PCR amplifications. Following primer sets were used for analyzing the expression of specific genes including FOXM1, forward: 5′-GGAGGAAATGCCACACTTAGC G-3′ and reverse: 5′-TAGGACTTCTTGGGTCTTGGGGTG-3′ as well as GAPDH, forward: 5′-GACCACAGTCCATGCCATCAC-3′ and reverse 5′-TCCACCACCCT GTTGCTGTAG-3′.

### Western blotting

For total lysate preparation, cells were lysed in RIPA buffer (50 mM Tris-HCl, 150 mM NaCl, 0.1% sodium dodecyl sulfate, and 1% Nonidet P-40 (NP-40); pH 7.4) supplemented with proteinase inhibitors (complete proteinase inhibitors, Roche, Indianapolis, IN, USA). Protein lysates (30 *μ*g per lane) were separated by 10% sodium dodecyl sulfate-polyacrylamide gel electrophoresis and processed for immunoblotting with antibodies against cleaved PARP (Enogene, Atlanta, GA, USA, E11-0365L), CD44 (GeneTex, Irvine, CA, USA, CTX102111), ALDH1 (GeneTex, GTX123973), Nanog (GeneTex, GTX100863), Oct4 (GeneTex, GTX101497), Bmi1 (GeneTex, GTX114008), Snail (Abcam, Cambridge, UK, Ab78105), Twist (GeneTex, GTX127310), FoxM1 (GeneTex, GTX100276), and Tubulin (Enogene, E1C601), respectively. Signals were detected using an enhanced chemiluminescence system (ECL, NEN Life Science, Boston, MA, USA).

## Figures and Tables

**Table 1:** IC_50_ values of oxaliplatin and thiostrepton on three human colon cancer cell lines

**Drug**	**Oxaliplatin (*μ*M)**	**Thiostrepton (*μ*M)**
*Cell line*		
HCT-15	24.3±3.2	5.1±0.7
HT-29	27.1±4.3	4.4±0.6
HCT-116	15.3±2.5	1.2±0.2

Cytotoxicity of oxaliplatin and thiostrepton on HCT-15, HT-29 and HCT-116 human colon cancer cells was analyzed by MTT assays and IC_50_ values were calculated

## Abbreviations

ALDH: aldehyde dehydrogenase
ARE: antioxidant response element
CRC: colorectal cancer
CRSCs: colorectal cancer stem cells
CMap: Connectivity Map
DMSO: dimethyl sulfoxide
ECL: enhanced chemiluminescence system
EMT: epithelial-to-mesenchymal transition
FoxM1: Forkhead box M1
NP-40: Nonidet P-40
Nrf2: nuclear factor-erythroid 2 p45-related factor 2
polyHEMA: poly-2-hydroxyethyl methacrylate
SC: stem cell

## References

[bib1] 1Brenner H, Kloor M, Pox CP. Colorectal cancer. Lancet 2014; 383: 1490–1502. 24225001 10.1016/S0140-6736(13)61649-9

[bib2] 2Siegel R, Desantis C, Jemal A. Colorectal cancer statistics 2014 CA Cancer J Clin 2014; 64: 104–117. 24639052 10.3322/caac.21220

[bib3] 3Jordan CT, Guzman ML, Noble M. Cancer stem cells. N Engl J Med 2006; 355: 1253–1261. 16990388 10.1056/NEJMra061808

[bib4] 4Charafe-Jauffret E, Ginestier C, Iovino F, Wicinski J, Cervera N, Finetti P et al. Breast cancer cell lines contain functional cancer stem cells with metastatic capacity and a distinct molecular signature. Cancer Res 2009; 69: 1302–1313. 19190339 10.1158/0008-5472.CAN-08-2741 PMC2819227

[bib5] 5O'Brien CA, Pollett A, Gallinger S, Dick JE. A human colon cancer cell capable of initiating tumour growth in immunodeficient mice. Nature 2007; 445: 106–110. 17122772 10.1038/nature05372

[bib6] 6Ricci-Vitiani L, Lombardi DG, Pilozzi E, Biffoni M, Todaro M, Peschle C et al. Identification and expansion of human colon-cancer-initiating cells. Nature 2007; 445: 111–115. 17122771 10.1038/nature05384

[bib7] 7Chu P, Clanton DJ, Snipas TS, Lee J, Mitchell E, Nguyen ML et al. Characterization of a subpopulation of colon cancer cells with stem cell-like properties. Int J Cancer 2009; 124: 1312–1321. 19072981 10.1002/ijc.24061

[bib8] 8Dalerba P, Dylla SJ, Park IK, Liu R, Wang X, Cho RW et al. Phenotypic characterization of human colorectal cancer stem cells. Proc Natl Acad Sci USA 2007; 104: 10158–10163. 17548814 10.1073/pnas.0703478104 PMC1891215

[bib9] 9Pang R, Law WL, Chu AC, Poon JT, Lam CS, Chow AK et al. A subpopulation of CD26+ cancer stem cells with metastatic capacity in human colorectal cancer. Cell Stem Cell 2010; 6: 603–615. 20569697 10.1016/j.stem.2010.04.001

[bib10] 10Huang EH, Hynes MJ, Zhang T, Ginestier C, Dontu G, Appelman H et al. Aldehyde dehydrogenase 1 is a marker for normal and malignant human colonic stem cells (SC) and tracks SC overpopulation during colon tumorigenesis. Cancer Res 2009; 69: 3382–3389. 19336570 10.1158/0008-5472.CAN-08-4418 PMC2789401

[bib11] 11Haraguchi N, Utsunomiya T, Inoue H, Tanaka F, Mimori K, Barnard GF et al. Characterization of a side population of cancer cells from human gastrointestinal system. Stem Cells 2006; 24: 506–513. 16239320 10.1634/stemcells.2005-0282

[bib12] 12Singh SK, Clarke ID, Terasaki M, Bonn VE, Hawkins C, Squire J et al. Identification of a cancer stem cell in human brain tumors. Cancer Res 2003; 63: 5821–5828. 14522905

[bib13] 13Dylla SJ, Beviglia L, Park IK, Chartier C, Raval J, Ngan L et al. Colorectal cancer stem cells are enriched in xenogeneic tumors following chemotherapy. PLoS One 2008; 3: e2428. 18560594 10.1371/journal.pone.0002428 PMC2413402

[bib14] 14Mani SA, Guo W, Liao MJ, Eaton EN, Ayyanan A, Zhou AY et al. The epithelial-mesenchymal transition generates cells with properties of stem cells. Cell 2008; 133: 704–715. 18485877 10.1016/j.cell.2008.03.027 PMC2728032

[bib15] 15van Es JH, Clevers H. Notch and Wnt inhibitors as potential new drugs for intestinal neoplastic disease. Trends Mol Med 2005; 11: 496–502. 16214417 10.1016/j.molmed.2005.09.008

[bib16] 16You L, He B, Xu Z, Uematsu K, Mazieres J, Fujii N et al. An anti-Wnt-2 monoclonal antibody induces apoptosis in malignant melanoma cells and inhibits tumor growth. Cancer Res 2004; 64: 5385–5389. 15289346 10.1158/0008-5472.CAN-04-1227

[bib17] 17Liu J, Pan S, Hsieh MH, Ng N, Sun F, Wang T et al. Targeting Wnt-driven cancer through the inhibition of Porcupine by LGK974. Proc Natl Acad Sci USA 2013; 110: 20224–20229. 24277854 10.1073/pnas.1314239110 PMC3864356

[bib18] 18Sandhiya S, Melvin G, Kumar SS, Dkhar SA. The dawn of hedgehog inhibitors: Vismodegib. J Pharmacol Pharmacother 2013; 4: 4–7. 23662017 10.4103/0976-500X.107628 PMC3643342

[bib19] 19Sahebjam S, Bedard PL, Castonguay V, Chen Z, Reedijk M, Liu G et al. A phase I study of the combination of ro4929097 and cediranib in patients with advanced solid tumours (PJC-004/NCI 8503). Br J Cancer 2013; 109: 943–949. 23868004 10.1038/bjc.2013.380 PMC3749563

[bib20] 20Marangoni E, Lecomte N, Durand L, de Pinieux G, Decaudin D, Chomienne C et al. CD44 targeting reduces tumour growth and prevents post-chemotherapy relapse of human breast cancers xenografts. Br J Cancer 2009; 100: 918–922. 19240712 10.1038/sj.bjc.6604953 PMC2661796

[bib21] 21Jin L, Hope KJ, Zhai Q, Smadja-Joffe F, Dick JE. Targeting of CD44 eradicates human acute myeloid leukemic stem cells. Nat Med 2006; 12: 1167–1174. 16998484 10.1038/nm1483

[bib22] 22Gupta PB, Onder TT, Jiang G, Tao K, Kuperwasser C, Weinberg RA et al. Identification of selective inhibitors of cancer stem cells by high-throughput screening. Cell 2009; 138: 645–659. 19682730 10.1016/j.cell.2009.06.034 PMC4892125

[bib23] 23Dallas NA, Xia L, Fan F, Gray MJ, Gaur P, van Buren G 2nd et al. Chemoresistant colorectal cancer cells, the cancer stem cell phenotype, and increased sensitivity to insulin-like growth factor-I receptor inhibition. Cancer Res 2009; 69: 1951–1957. 19244128 10.1158/0008-5472.CAN-08-2023 PMC3198868

[bib24] 24Ramirez A, Boulaiz H, Morata-Tarifa C, Peran M, Jimenez G, Picon-Ruiz M et al. HER2-signaling pathway, JNK and ERKs kinases, and cancer stem-like cells are targets of Bozepinib small compound. Oncotarget 2014; 5: 3590–3606. 24946763 10.18632/oncotarget.1962 PMC4116505

[bib25] 25Todaro M, Alea MP, Di Stefano AB, Cammareri P, Vermeulen L, Iovino F et al. Colon cancer stem cells dictate tumor growth and resist cell death by production of interleukin-4. Cell Stem Cell 2007; 1: 389–402. 18371377 10.1016/j.stem.2007.08.001

[bib26] 26Lombardo Y, Scopelliti A, Cammareri P, Todaro M, Iovino F, Ricci-Vitiani L et al. Bone morphogenetic protein 4 induces differentiation of colorectal cancer stem cells and increases their response to chemotherapy in mice. Gastroenterology 2011; 140: 297–309. 20951698 10.1053/j.gastro.2010.10.005

[bib27] 27Cai Z, Ke J, He X, Yuan R, Chen Y, Wu X et al. Significance of mTOR signaling and its inhibitor against cancer stem-like cells in colorectal cancer. Ann Surg Oncol 2013; 21: 179–188. 23907312 10.1245/s10434-013-3146-8

[bib28] 28Qu XA, Rajpal DK. Applications of Connectivity Map in drug discovery and development. Drug Discov Today 2012; 17: 1289–1298. 22889966 10.1016/j.drudis.2012.07.017

[bib29] 29Gartel AL. FoxM1 inhibitors as potential anticancer drugs. Expert Opin Ther Targets 2008; 12: 663–665. 18479213 10.1517/14728222.12.6.663

[bib30] 30Radhakrishnan SK, Bhat UG, Hughes DE, Wang IC, Costa RH, Gartel AL. Identification of a chemical inhibitor of the oncogenic transcription factor forkhead box M1. Cancer Res 2006; 66: 9731–9735. 17018632 10.1158/0008-5472.CAN-06-1576

[bib31] 31Lamb J, Crawford ED, Peck D, Modell JW, Blat IC, Wrobel MJ et al. The Connectivity Map: using gene-expression signatures to connect small molecules, genes, and disease. Science 2006; 313: 1929–1935. 17008526 10.1126/science.1132939

[bib32] 32Suva ML, Riggi N, Janiszewska M, Radovanovic I, Provero P, Stehle JC et al. EZH2 is essential for glioblastoma cancer stem cell maintenance. Cancer Res 2009; 69: 9211–9218. 19934320 10.1158/0008-5472.CAN-09-1622

[bib33] 33Yang AD, Fan F, Camp ER, van Buren G, Liu W, Somcio R et al. Chronic oxaliplatin resistance induces epithelial-to-mesenchymal transition in colorectal cancer cell lines. Clin Cancer Res 2006; 12: 4147–4153. 16857785 10.1158/1078-0432.CCR-06-0038

[bib34] 34Dean M, Fojo T, Bates S. Tumour stem cells and drug resistance. Nat Rev Cancer 2005; 5: 275–284. 15803154 10.1038/nrc1590

[bib35] 35Haraguchi N, Ohkuma M, Sakashita H, Matsuzaki S, Tanaka F, Mimori K et al. CD133+CD44+ population efficiently enriches colon cancer initiating cells. Ann Surg Oncol 2008; 15: 2927–2933. 18663533 10.1245/s10434-008-0074-0

[bib36] 36Bhat UG, Zipfel PA, Tyler DS, Gartel AL. Novel anticancer compounds induce apoptosis in melanoma cells. Cell Cycle 2008; 7: 1851–1855. 18583930 10.4161/cc.7.12.6032

[bib37] 37Uddin S, Ahmed M, Hussain A, Abubaker J, Al-Sanea N, AbdulJabbar A et al. Genome-wide expression analysis of Middle Eastern colorectal cancer reveals FOXM1 as a novel target for cancer therapy. Am J Pathol 2011; 178: 537–547. 21281787 10.1016/j.ajpath.2010.10.020 PMC3070566

[bib38] 38Bhat UG, Halasi M, Gartel AL. FoxM1 is a general target for proteasome inhibitors. PLoS One 2009; 4: e6593. 19672316 10.1371/journal.pone.0006593 PMC2721658

[bib39] 39Wierstra I. FOXM1 (Forkhead box M1) in tumorigenesis: overexpression in human cancer, implication in tumorigenesis, oncogenic functions, tumor-suppressive properties, and target of anticancer therapy. Adv Cancer Res 2013; 119: 191–419. 23870513 10.1016/B978-0-12-407190-2.00016-2

[bib40] 40Chiu WT, Huang YF, Tsai HY, Chen CC, Chang CH, Huang SC et al. FOXM1 confers to epithelial-mesenchymal transition, stemness and chemoresistance in epithelial ovarian carcinoma cells. Oncotarget 2015; 6: 2349–2365. 25537512 10.18632/oncotarget.2957 PMC4385856

[bib41] 41Bergamaschi A, Madak-Erdogan Z, Kim YJ, Choi YL, Lu H, Katzenellenbogen BS. The forkhead transcription factor FOXM1 promotes endocrine resistance and invasiveness in estrogen receptor-positive breast cancer by expansion of stem-like cancer cells. Breast Cancer Res 2014; 16: 436. 25213081 10.1186/s13058-014-0436-4 PMC4303117

[bib42] 42Myatt SS, Lam EW. The emerging roles of forkhead box (Fox) proteins in cancer. Nat Rev Cancer 2007; 7: 847–859. 17943136 10.1038/nrc2223

[bib43] 43Xie Z, Tan G, Ding M, Dong D, Chen T, Meng X et al. Foxm1 transcription factor is required for maintenance of pluripotency of P19 embryonal carcinoma cells. Nucleic Acids Res 2010; 38: 8027–8038. 20702419 10.1093/nar/gkq715 PMC3001083

[bib44] 44Joshi K, Banasavadi-Siddegowda Y, Mo X, Kim SH, Mao P, Kig C et al. MELK-dependent FOXM1 phosphorylation is essential for proliferation of glioma stem cells. Stem Cells 2013; 31: 1051–1063. 23404835 10.1002/stem.1358 PMC3744761

[bib45] 45Visnyei K, Onodera H, Damoiseaux R, Saigusa K, Petrosyan S, De Vries D et al. A molecular screening approach to identify and characterize inhibitors of glioblastoma stem cells. Mol Cancer Ther 2011; 10: 1818–1828. 21859839 10.1158/1535-7163.MCT-11-0268 PMC3191241

[bib46] 46Wang IC, Chen YJ, Hughes D, Petrovic V, Major ML, Park HJ et al. Forkhead box M1 regulates the transcriptional network of genes essential for mitotic progression and genes encoding the SCF (Skp2-Cks1) ubiquitin ligase. Mol Cell Biol 2005; 25: 10875–10894. 16314512 10.1128/MCB.25.24.10875-10894.2005 PMC1316960

[bib47] 47Bao B, Wang Z, Ali S, Kong D, Banerjee S, Ahmad A et al. Over-expression of FoxM1 leads to epithelial-mesenchymal transition and cancer stem cell phenotype in pancreatic cancer cells. J Cell Biochem 2011; 112: 2296–2306. 21503965 10.1002/jcb.23150 PMC3155646

[bib48] 48Halasi M, Schraufnagel DP, Gartel AL. Wild-type p53 protects normal cells against apoptosis induced by thiostrepton. Cell Cycle 2009; 8: 2850–2851. 19652535 10.4161/cc.8.17.9414

[bib49] 49Bhat UG, Halasi M, Gartel AL. Thiazole antibiotics target FoxM1 and induce apoptosis in human cancer cells. PLoS One 2009; 4: e5592. 19440351 10.1371/journal.pone.0005592 PMC2680058

[bib50] 50Pilarsky C, Wenzig M, Specht T, Saeger HD, Grutzmann R. Identification and validation of commonly overexpressed genes in solid tumors by comparison of microarray data. Neoplasia 2004; 6: 744–750. 15720800 10.1593/neo.04277 PMC1531678

[bib51] 51Liu M, Dai B, Kang SH, Ban K, Huang FJ, Lang FF et al. FoxM1B is overexpressed in human glioblastomas and critically regulates the tumorigenicity of glioma cells. Cancer Res 2006; 66: 3593–3602. 16585184 10.1158/0008-5472.CAN-05-2912

[bib52] 52Dai B, Kang SH, Gong W, Liu M, Aldape KD, Sawaya R et al. Aberrant FoxM1B expression increases matrix metalloproteinase-2 transcription and enhances the invasion of glioma cells. Oncogene 2007; 26: 6212–6219. 17404569 10.1038/sj.onc.1210443

[bib53] 53Huang C, Qiu Z, Wang L, Peng Z, Jia Z, Logsdon CD et al. A novel FoxM1-caveolin signaling pathway promotes pancreatic cancer invasion and metastasis. Cancer Res 2012; 72: 655–665. 22194465 10.1158/0008-5472.CAN-11-3102 PMC3271134

[bib54] 54Li Q, Zhang N, Jia Z, Le X, Dai B, Wei D et al. Critical role and regulation of transcription factor FoxM1 in human gastric cancer angiogenesis and progression. Cancer Res 2009; 69: 3501–3509. 19351851 10.1158/0008-5472.CAN-08-3045 PMC4087044

[bib55] 55Zhang N, Wei P, Gong A, Chiu WT, Lee HT, Colman H et al. FoxM1 promotes beta-catenin nuclear localization and controls Wnt target-gene expression and glioma tumorigenesis. Cancer Cell 2011; 20: 427–442. 22014570 10.1016/j.ccr.2011.08.016 PMC3199318

[bib56] 56Carr JR, Park HJ, Wang Z, Kiefer MM, Raychaudhuri P. FoxM1 mediates resistance to herceptin and paclitaxel. Cancer Res 2010; 70: 5054–5063. 20530690 10.1158/0008-5472.CAN-10-0545 PMC2893542

[bib57] 57Kwok JM, Peck B, Monteiro LJ, Schwenen HD, Millour J, Coombes RC et al. FOXM1 confers acquired cisplatin resistance in breast cancer cells. Mol Cancer Res 2010; 8: 24–34. 20068070 10.1158/1541-7786.MCR-09-0432 PMC2809047

[bib58] 58Mraz M, Zent CS, Church AK, Jelinek DF, Wu X, Pospisilova S et al. Bone marrow stromal cells protect lymphoma B-cells from rituximab-induced apoptosis and targeting integrin alpha-4-beta-1 (VLA-4) with natalizumab can overcome this resistance. Br J Haematol 2011; 155: 53–64. 21749361 10.1111/j.1365-2141.2011.08794.x PMC4405035

[bib59] 59Akhdar H, Loyer P, Rauch C, Corlu A, Guillouzo A, Morel F. Involvement of Nrf2 activation in resistance to 5-fluorouracil in human colon cancer HT-29 cells. Eur J Cancer 2009; 45: 2219–2227. 19524433 10.1016/j.ejca.2009.05.017

[bib60] 60Barrett T, Wilhite SE, Ledoux P, Evangelista C, Kim IF, Tomashevsky M et al. NCBI GEO: archive for functional genomics data sets—update. Nucleic Acids Res 2013; 41: D991–D995. 23193258 10.1093/nar/gks1193 PMC3531084

[bib61] 61Ju SY, Chiou SH, Su Y. Maintenance of the stemness in CD44(+) HCT-15 and HCT-116 human colon cancer cells requires miR-203 suppression. Stem Cell Res 2014; 12: 86–100. 24145190 10.1016/j.scr.2013.09.011

[bib62] 62Ghosh S, Spagnoli GC, Martin I, Ploegert S, Demougin P, Heberer M et al. Three-dimensional culture of melanoma cells profoundly affects gene expression profile: a high density oligonucleotide array study. J Cell Physiol 2005; 204: 522–531. 15744745 10.1002/jcp.20320

