# Supplementary material for: Identification of thiostrepton as a novel therapeutic agent that targets human colon cancer stem cells
Source: Cell Death Dis. 2015 Jul 2;6(7):e1801–. doi: 10.1038/cddis.2015.155 (PMC4650716; doi:10.1038/cddis.2015.155)
Supplement: Supplementary Information [file cddis2015155x1.ppt]

## Slide 1
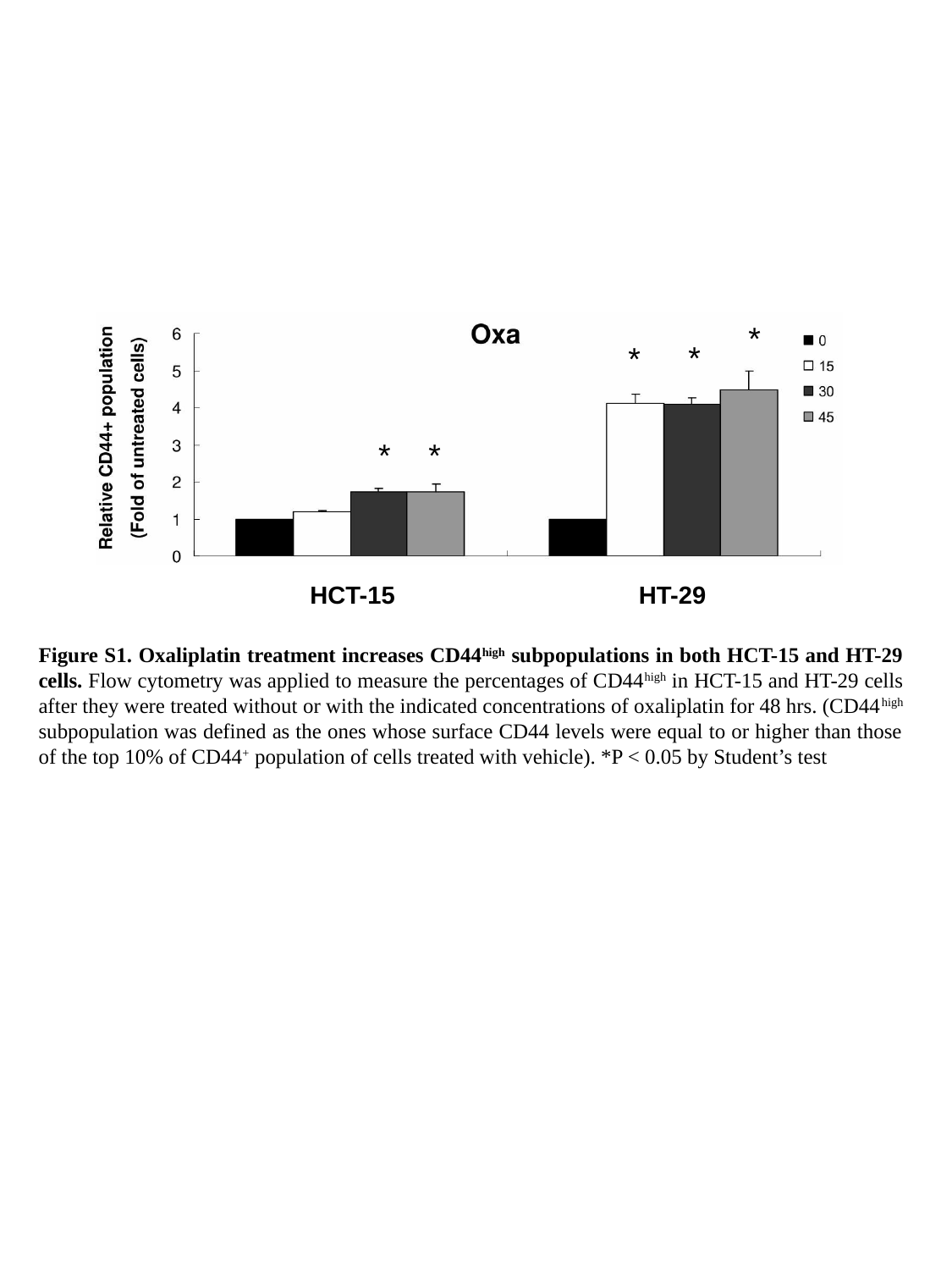

*
*
*
*
*
 HCT-15 HT-29
Figure S1. Oxaliplatin treatment increases CD44high subpopulations in both HCT-15 and HT-29 cells. Flow cytometry was applied to measure the percentages of CD44high in HCT-15 and HT-29 cells after they were treated without or with the indicated concentrations of oxaliplatin for 48 hrs. (CD44high subpopulation was defined as the ones whose surface CD44 levels were equal to or higher than those of the top 10% of CD44+ population of cells treated with vehicle). *P < 0.05 by Student’s test

## Slide 2
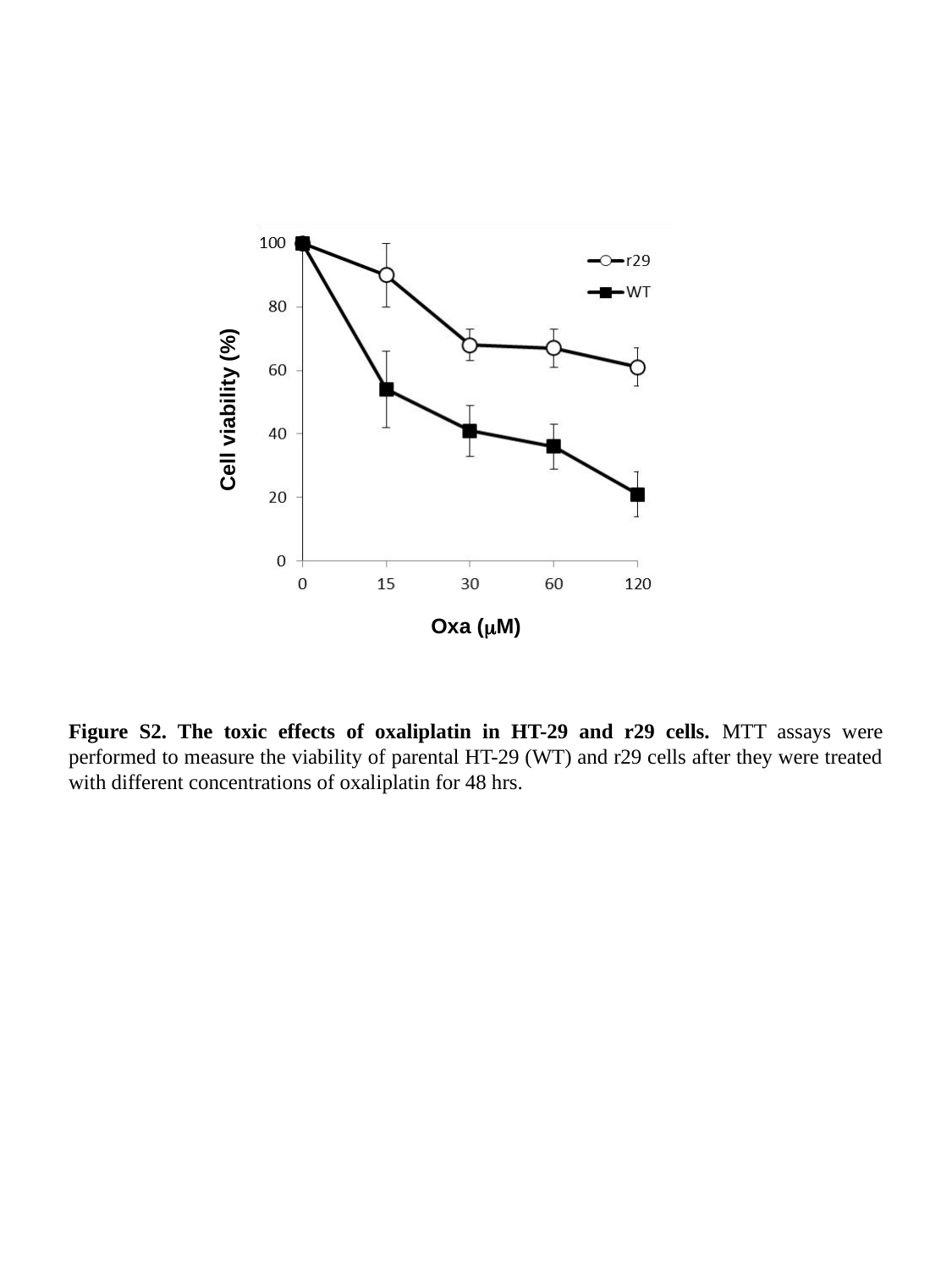

Cell viability (%)
Oxa (μM)
Figure S2. The toxic effects of oxaliplatin in HT-29 and r29 cells. MTT assays were performed to measure the viability of parental HT-29 (WT) and r29 cells after they were treated with different concentrations of oxaliplatin for 48 hrs.

## Slide 3
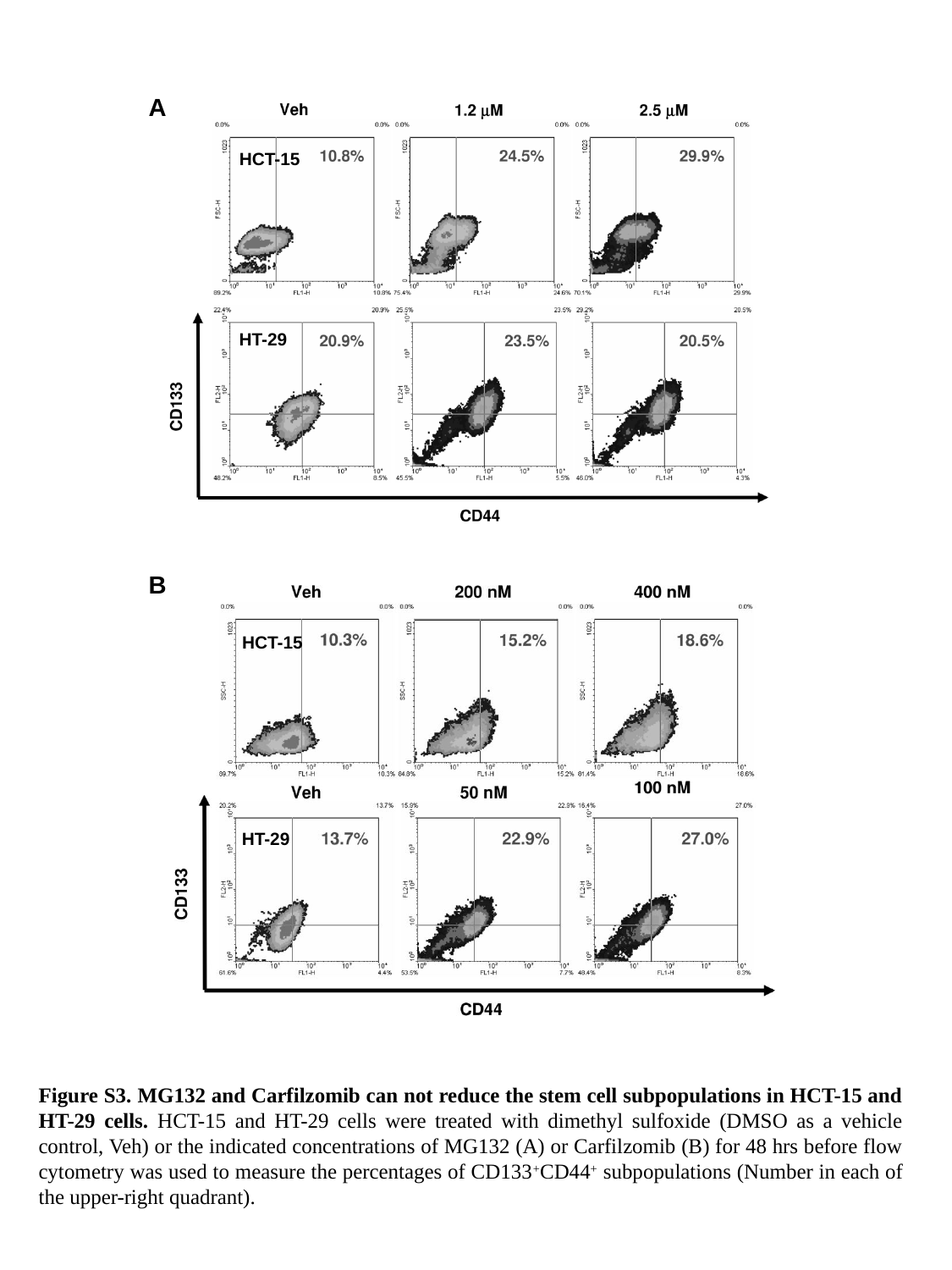

A
HCT-15
HT-29
B
HCT-15
HT-29
Figure S3. MG132 and Carfilzomib can not reduce the stem cell subpopulations in HCT-15 and HT-29 cells. HCT-15 and HT-29 cells were treated with dimethyl sulfoxide (DMSO as a vehicle control, Veh) or the indicated concentrations of MG132 (A) or Carfilzomib (B) for 48 hrs before flow cytometry was used to measure the percentages of CD133+CD44+ subpopulations (Number in each of the upper-right quadrant).

## Slide 4
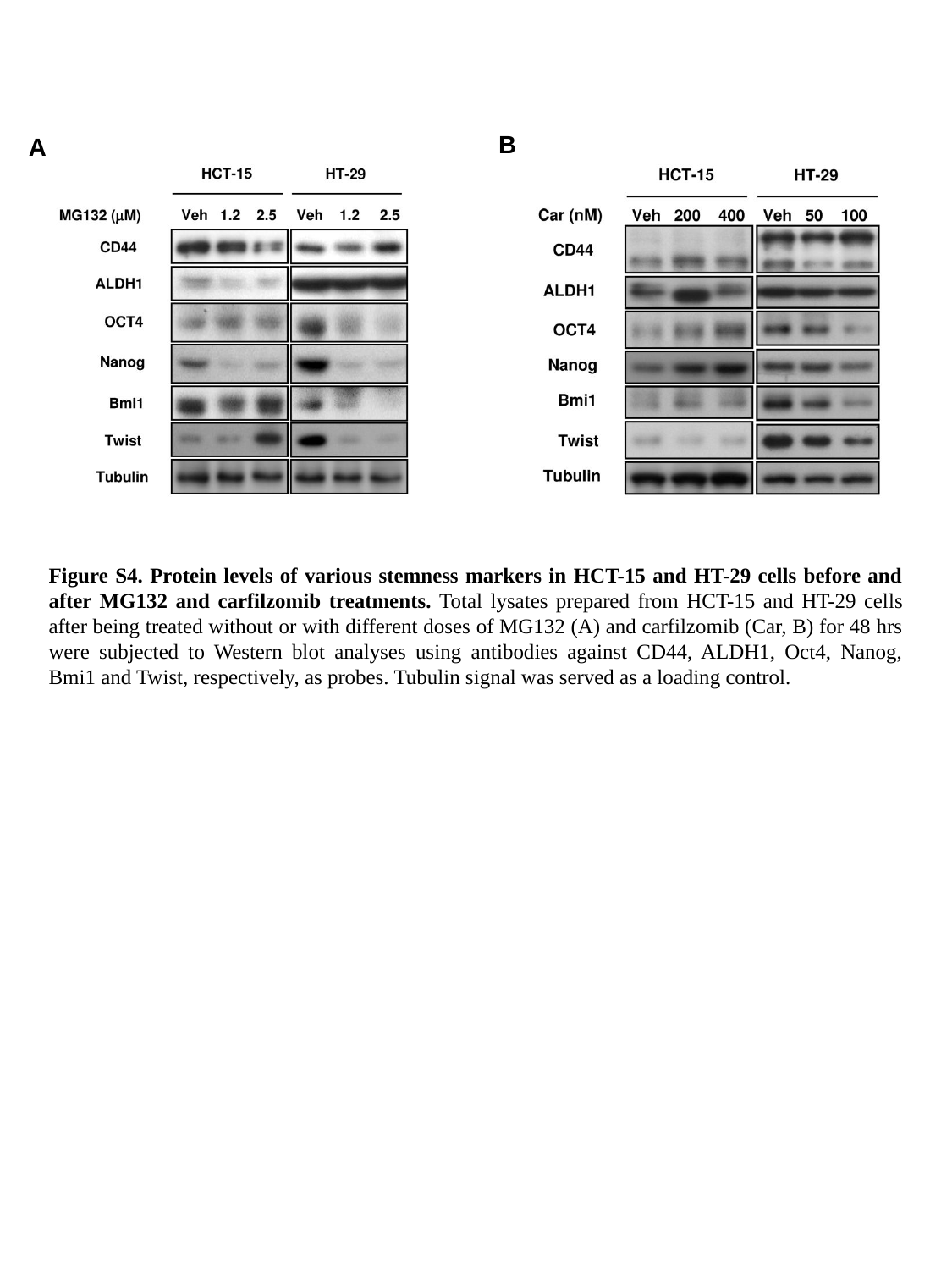

B
A
Figure S4. Protein levels of various stemness markers in HCT-15 and HT-29 cells before and after MG132 and carfilzomib treatments. Total lysates prepared from HCT-15 and HT-29 cells after being treated without or with different doses of MG132 (A) and carfilzomib (Car, B) for 48 hrs were subjected to Western blot analyses using antibodies against CD44, ALDH1, Oct4, Nanog, Bmi1 and Twist, respectively, as probes. Tubulin signal was served as a loading control.
